# Identification and characterization of L1-specific endo-siRNAs essential for early embryonic development in pig

**DOI:** 10.18632/oncotarget.15517

**Published:** 2017-02-19

**Authors:** Heng Zhang, Jilong Liu, Yurong Tai, Xiaolei Zhang, Jiaming Zhang, Shichao Liu, Jiawei Lv, Zhonghua Liu, Qingran Kong

**Affiliations:** ^1^ Laboratory of Embryo Biotechnology, College of Life Science, Northeast Agricultural University, Harbin, Heilongjiang Province 150030, China; ^2^ College of Veterinary Medicine, South China Agricultural University, Guangzhou Province 510642, China

**Keywords:** endo-siRNA, deep sequencing, L1, early embryonic development, pig

## Abstract

Small noncoding RNAs (sncRNAs) play important roles in RNA interference (RNAi). In addition to microRNA (miRNA) and Piwi-interacting RNA (piRNA), one key member of sncRNAs group is endogenous small interfering RNA (endo-siRNA). Some studies do show the role of endo-siRNAs in Dicer and/or Ago mutants, however, the biological functions of specific endo-siRNAs remains mostly unanswered. In the study, we have performed a comparative analysis of endo-siRNAs present in porcine sperms, oocytes and zygotes, identified by deep sequencing and bioinformatics analysis. Further, we observe a large amount of endo-siRNAs specific binding on ORF2 and 3′ UTR of porcine L1 (L1-siRNAs). And, 9 L1-siRNAs generated from a dsRNA formed between L1 transcript and a newly identified an antisense noncoding RNA was characterized. We show the L1-siRNAs regulate early embryonic development by inhibiting the activity of L1 retrotransposition. This work can contribute to understanding the functional role of abundant endo-siRNAs in embryonic development.

## INTRODUCTION

RNA interference (RNAi) is a sequence-dependent mechanism in gene regulation [1]. The mechanism is determined by a family of small noncoding RNAs (sncRNAs), including microRNA (miRNA), Piwi-interacting RNA (piRNA) and endogenous small interfering RNA (endo-siRNA) [2, 3]. In RNAi, endo-siRNA, with a length of 18–24 nt, has evidenced to play key roles. Endo-siRNAs are processed from long double-stranded RNA (dsRNA) precursors by digestion with an RNase III enzyme, DICER, and then one of the two strands guides AGO2,the endonucleolytic component of RNA-induced silencing complex (RISC), to cleave the targets [4, 5]. Endo-siRNAs are perfectly complementary to their targets and trigger pre-mRNA/mRNA cleavage. The structures of the dsRNA precursors of endo-siRNAs are derived from transposable elements, complementary annealed transcripts, and long “fold-back” transcripts called hairpin RNAs (hpRNAs) [5–7]. According to the dsRNA structures, endo-siRNAs can be characterized.

Generally, endo-siRNAs are mainly involved in defense against viruses, transposons, and transgenes through RNAi [8]. Recently, in germ cells and early embryos, it has been ascribed a functional role. In mouse, oocytes with deficient siRNA pathway fail to complete meiosis I, and display severe spindle formation and chromosome alignment defects [9, 10]. Moreover, Wu et al. generated male germ line-specific Dicer conditional KO mice and found Dicer KO males with low sperm counts, low sperm motility and abnormal sperm morphology [11]. And, embryos derived from the endo-siRNAs-deficient sperm by intra cytoplasmic sperm injection (ICSI) displayed developmental failure [12]. Although these studies have demonstrated that endo-siRNAs are essential for gametogenesis and preimplantation embryonic development, little is known about the biogenesis and function of specific endo-siRNAs. And, the number of endo-siRNAs identified so far is few, only in mouse gametes and cultured human cells [13–15].So more endo-siRNAs needs to be identified and the function of specific endo-siRNA needs to be determined during special biological processes.

In the study, endo-siRNAs present in porcine sperms, oocytes and zygotes were identified by high-throughput sequencing and bioinformatics analysis. Three subclasses of endo-siRNAs were classified: transposable elements-associated siRNAs (TEs-siRNAs), inverted-complement mRNA-associated siRNAs (IC-siRNAs) and long hairpin RNA-associated siRNAs (Lhp-siRNAs). To clarify the function of endo-siRNAs in early embryos, we focused on the high-expressed endo-siRNAs in zygotes and found a large amount of endo-siRNAs specific binding on ORF2 and 3′ UTR of porcine L1 (L1-siRNAs). Furthermore, we evidenced that 9 L1-siRNAs were produced from dsRNA formed between L1 transcript and an antisense noncoding RNA, asL1, by an antisense promoter (ASP) in the L1 5′UTR, and could regulate early embryonic development by inhibiting L1 retrotransposon activity.

## RESULTS

### Identification of endo-siRNAs in porcine sperm, oocyte and zygote

**Figure 1:**
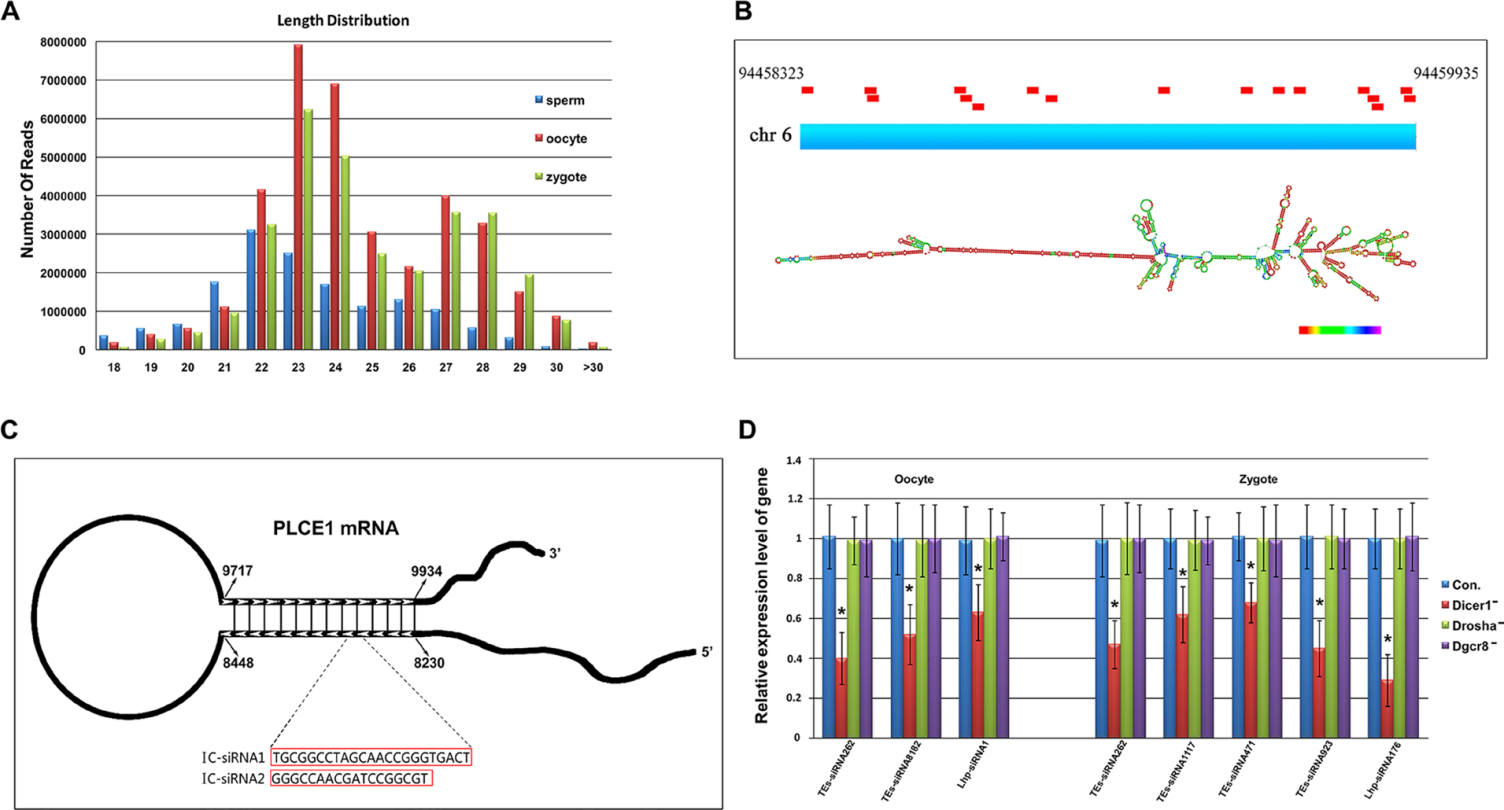
Identification of endo-siRNAs in porcine sperms, oocytes and zygotes (**A**) Length distribution of total small RNAs; (**B**) Structure of the IC-siRNAs cluster at Plce1 mRNA; (**C**) Structure of the Lhp-siRNAs cluster at the chr 6 locus. The Lhp-siRNAs mapped in this region are represented by red bars; (**D**) Expression changes of endo-siRNAs in Dicer1, Drosha, Dgcr8 knockdown oocytes and zygotes. Error bars represent s.d. (*n* = 3). *indicates *p* < 0.001.

Libraries from sncRNAs were prepared from 5 × 10^9^ sperms, 7845 oocytes and 6800 zygotes of pig and sequenced by high-throughput deep sequencing (llumina). We obtained 90431, 244480 and 219971 sncRNA sequences completely matched the pig genome from the sperms, oocytes and zygotes, respectively and the total number of reads was lower in sperms than those of oocytes and zygotes (Table 1). The length distribution of the total sncRNAs showed a bimodal pattern (Figure 1A): one peak was observed at 23nt, corresponding to the length of miRNAs and endo-siRNAs, and the other at 27–28nt, corresponding to the length of piRNAs [16]. Due to sncRNA populations have been less studied in pig, so the first challenge of the study was to classify the sncRNAs into miRNAs, piRNAs and endo-siRNAs. The piRNA population we annotated contained sequences that are homologous to the sequences of human and mouse piRNAs from piRNABank [17] plus sequences more than 24nt (Supplementary Table 1), and the miRNA population comprised sequences, with a length of 21–24nt [13], homogenous to the pig, human and mouse database [18, 19] (miRBase; Supplementary Table 2). The other 18–24nt sncRNAs were used to annotate endo-siRNAs. It has been reported that both piRNAs and endo-siRNAs can be associated with transposons and other genome repeated sequences [20–23]. Firstly, we identified endo-siRNAs associated with transposons. The sequences of sncRNAs matched to transposon elements, such as “LTR”,”SINE”,”L1”,and”ERV”, were characterized as TEs-siRNAs, and a large proportion of endo-siRNAs were in the subclass (Supplementary Table 3). To identify long hairpin RNA-associated siRNAs, we searched the genome region compassing more than 15 unique sncRNA sequences less than 10kb, and then we extracted the regions of every clusters to predict RNA secondary structure using RNAfold, and we characterized the endo-siRNAs mapped in the predicted long hairpin RNA as Lhp-siRNAs (Supplementary Table 3). One of the Lhp-siRNAs clusters observed in zygotes was located at the chromosome 6. In the cluster, 17 Lhp-siRNAs were mapped in a 1612 nt region (Figure 1B).By close inspection, we also found some sncRNAs mapped to inverted complement regions of mRNAs, and we characterized the subclass of endo-siRNAs derived from inverted-complement mRNA as IC-siRNAs (Supplementary Table 3). A cluster of IC-siRNAs from Plce1 mRNA was shown (Figure 1C). Plce1 mRNA has a length of 217 nt inverted complement region and 2 IC-siRNAs were observed in the region. Finally, we classified the total sequences and reads of sncRNA classes in Table 1. DICER, not DROSHA and DGCR8, is involved in the endo-siRNA biogenesis, so, to further confirm the result, we injected Locked Nucleic Acid (LNA) of Dicer1, Drosha and Dgcr8 in oocytes at the GV stage and detected the expressions of endo-siRNAs in MII oocytes and zygotes. The effective knockdown of Dicer1, Drosha, Dgcr8 were verified by Q-PCR (Supplementary Figure 1A) and, by Western blot, the knockdown of Dicer1 protein was confirmed (Supplementary Figure 1B). Furthermore, we checked the expression of 50 endo-siRNAs, and found the expression of 3 and 5 endo-siRNAs significantly decreased respectively in Dicer-knockdown oocytes and zygotes (*p* < 0.001) but had no significant changes in Drosha- or Dgcr8- knockdown groups (Figure 1D). Only a small part of endo-siRNAs tested were reduced, suggesting endo-siRNAs may be processed at the early stage of oocyte maturation before Dicer knockdown. Combined with the facts, we identify endo-siRNAs in porcine sperm, oocyte and zygote.

**Table 1:** Total sequences and reads of sncRNA classes classified

RNA class	Sperm		Oocyte		Zygote	
	Total sequences (%)	Total reads (%)	Total sequences (%)	Total reads (%)	Total sequences (%)	Total reads (%)
**miRNA**	665 (0.7)	4424759 (29)	556 (0.2)	2322725 (6)	492 (0.2)	1987289 (6)
**piRNA**	40833 (45)	4071893 (27)	91335 (37)	15128077 (42)	89854 (41)	14477019 (47)
**endo-siRNA**	1087 (1)	214410 (1)	9092 (4)	1899141 (5)	8574 (4)	2036187 (7)
**unknown**	47846 (53)	5282450 (35)	142960 (59)	16756775 (46)	121038 (55)	12188084 (40)
**total**	90431 (100)	15096401 (100)	244480 (100)	36271523 (100)	219971 (100)	30688579 (100)

### Endo-siRNAs derived from porcine L1 retrotransposon

**Figure 2:**
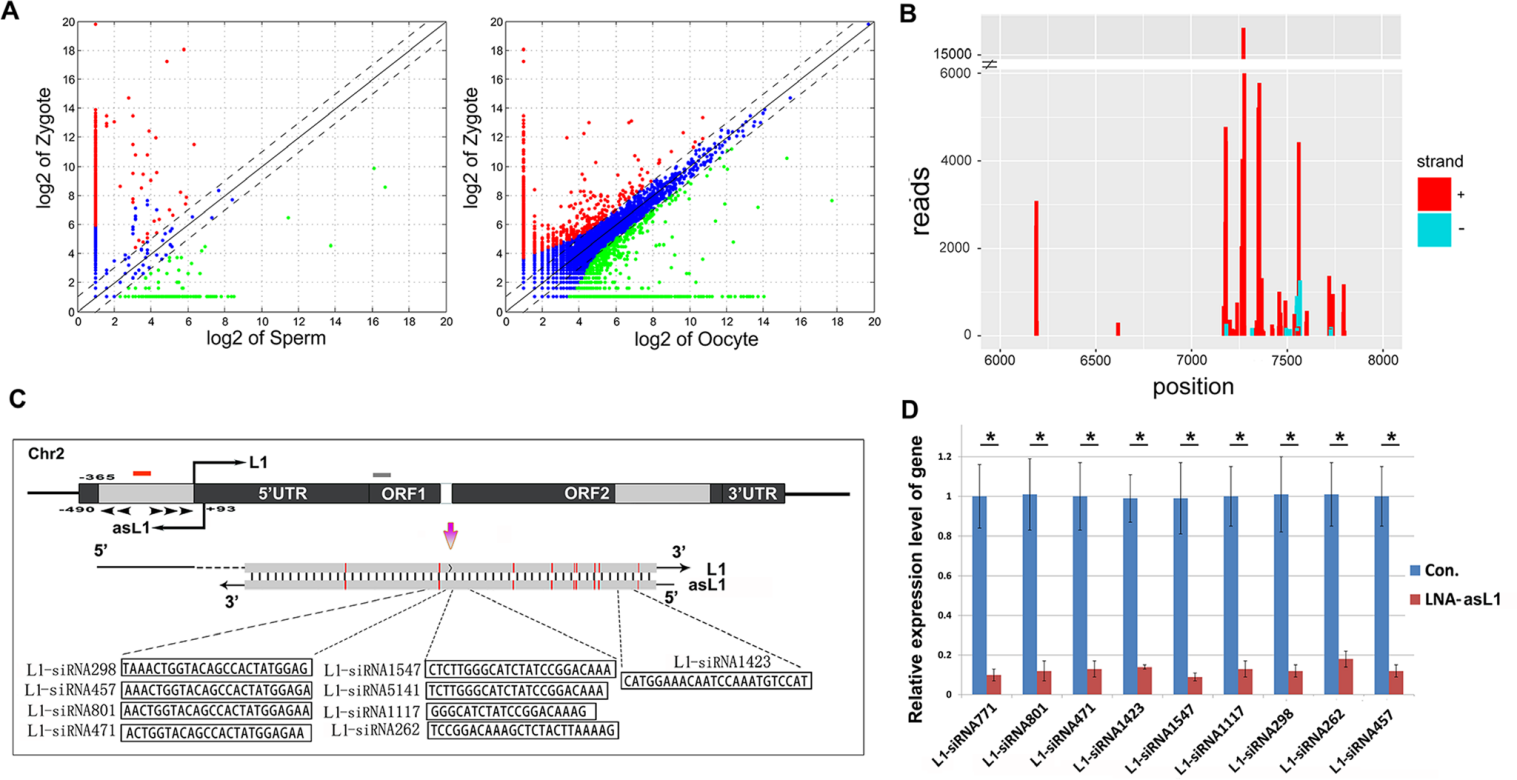
Endo-siRNAs derived from porcine L1 retrotransposon (**A**) Analysis of high expressed endo-siRNAs in zygotes compared to sperms and oocytes; (**B**) Read counts of L1-siRNAs and their binding sites on L1 retrotransposon. L1-siRNAs derived from sense and antisense strands of L1 were marked by red and blue; (**C**) L1-siRNAs generated from a dsRNA formed between asL1 and L1 ORF2. LNA-asL1 and LNA-L1 are represented by red and grey bars, respectively. Arrowheads represent primers used in 5′ and 3′ RACE. The base mismatch was marked by red; (**D**) Effective knockdown of L1-siRNAs by LNA-asL1. Error bars represent s.d. (*n* = 3). * indicates *p* < 0.001.

To clarify the function of endo-siRNAs during early embryonic development, we performed expression analysis of differential endo-siRNAs between zygote and sperm or oocyte, and focused on the high-expressed endo-siRNAs in zygotes (Figure 2A; FD > 2; *P value* < 0.01). Deep analysis showed many high-expressed TEs-siRNAs mapped to the ORF2 and 3′ UTR regions of porcine L1 retrotransposon (Figure 2B). We refer to these L1-specific endo-siRNAs as L1-siRNAs and elaborate the mechanism of formation. Natural antisense transcripts (NATs) can interfere with the expression of complementary sense transcripts with exquisite specificity [3, 24–26]. Furthermore, an ASP in the L1 5′ UTR has been reported in human [27], so we presume that the product of ASP could form dsRNA with L1 ORF2 and 3′ UTR regions and the L1-siRNAs are processed from the dsRNA by digestion with DICER. Thus, we extracted156 upstream sequences of 3000bp of L1 from pig genome and performed multiple sequence alignment with L1 ORF2 and 3′ UTR sequence by blast. Lots of homologous sequences were observed (Supplementary Figure 2A) and we interested in a sequence from L1 transcription start site to −365 on chromosome 2 which is homolog with a part of ORF2 sequence (Figure 2C). The transcription of the sequence was verified by RT-PCR in zygotes (Supplementary Figure 2B). To better understand the transcript, we detected its transcription start and end sites by 5′ and 3′ rapid amplification of complementary DNA ends (RACE). We found the transcript initiates at +93 and ends at −490 (Figure 2C).We observed only several mini open reading frames (ORFs)in the transcript by NCBI ORF Finder (Supplementary Figure 2C). Thus, the antisense transcript is a long noncoding RNA, and we named it as antisense L1 (asL1). 365bp dsRNA structure could be formed between L1 ORF2 and asL1, and 9 L1-siRNAs with high reads mapped to the region (Figure 2C). To confirm that, we performed asL1knockdown experiment to check expression changes of the L1-siRNAs. AsL1 was effectively knockdown by LNA-siRNA (LNA-asL1) in zygotes (Supplementary Figure 2D), and the expression of L1-siRNAs were significantly decreased (Figure 2D), indicating the 9 L1-siRNAs are generated from the dsRNA structure formed between L1 ORF2 and asL1.

### L1-siRNAs regulate early embryonic development

**Figure 3:**
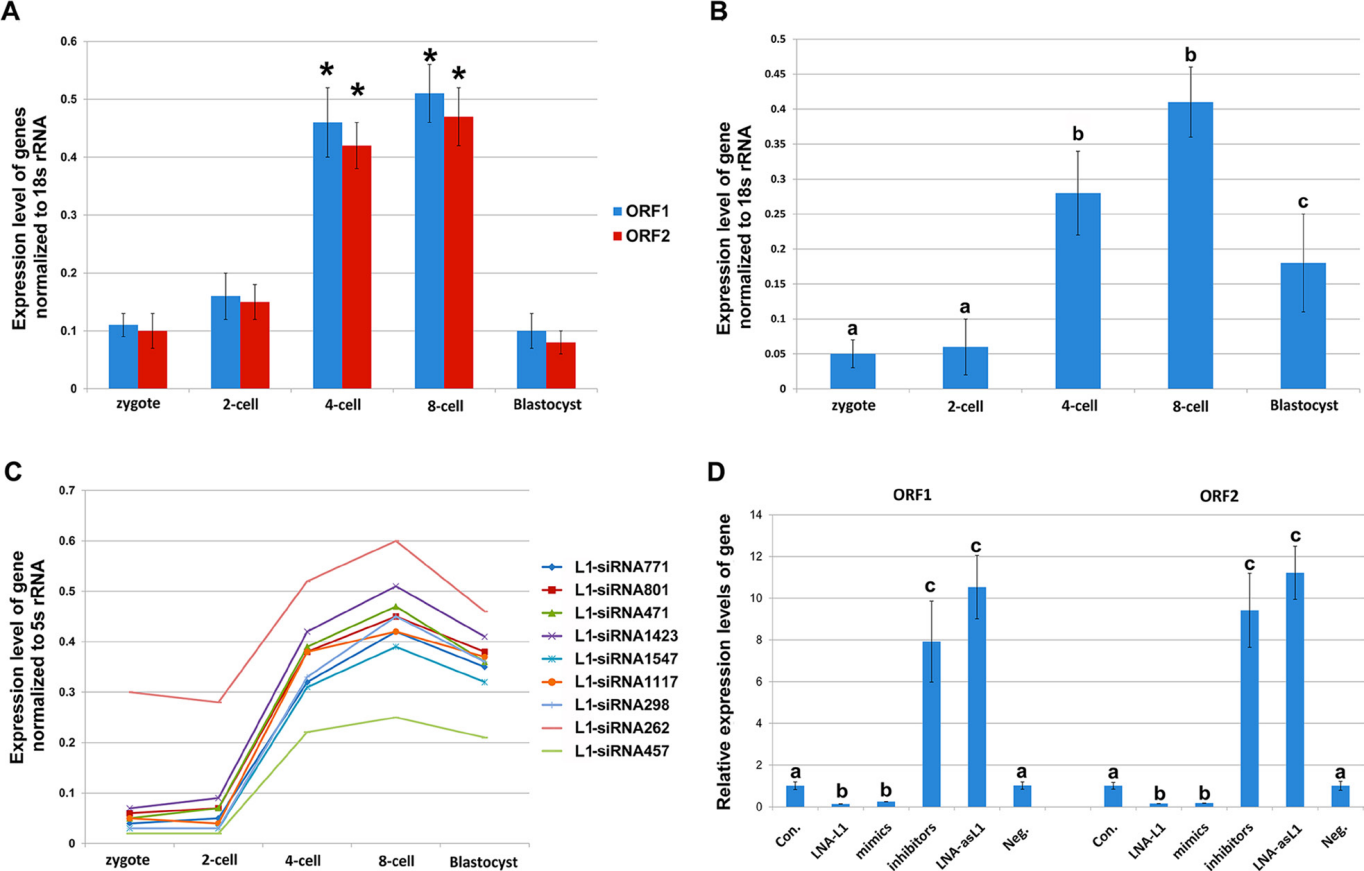
Expression of L1, asL1, and L1 -siRNAs during *in vitro* development of porcine IVF embryos (**A**) Expression of L1 ORF1 and ORF2 checked by Q-PCR. *indicates *p* < 0.001.; (**B**) Expression of asL1 checked by Q-PCR; (**C**) Expression of L1-siRNAs checked by Q-PCR. Values with different superscripts differ significantly (*p* < 0.001); (**D**) Expression changes of L1 ORF1 and ORF2 by LNA-L1, LNA-asL1, L1-siRNAs mimics and inhibitors. Values with different superscripts differ significantly (*p* < 0.001). Error bars represent s.d. (*n* = 3).

To investigate the role of L1-siRNAs during early embryonic development, we injected the mixture of L1-siRNAs mimics or inhibitors into MII oocytes and checked the *in vitro* developmental competency of porcine IVF embryos. The results showed that the rates of cleavage had no significant difference among every control and experimental groups, but the proportions of embryos that developed to blastocysts in mimics and inhibitors groups were significantly lower than those of embryos in control and neg. siRNA groups (2.24 and 8.51 versus 16.57 and 14.27%, respectively; *p* < 0.05; Table 2 and Supplementary Figure 3). Furthermore, the proportion of embryos developed to the blastocyst stage in LNA-asL1 group was significantly decreased compared to control and Neg. LNA group (6.08 versus 16.57and 17.35%; *p* < 0.05; Table 2 and Figure 3). And, there was no significant difference among mimics, inhibitors and LNA-asL1 groups in the blastocyst rates (*p* > 0.05). We also observed more mimics, inhibitors and LNA-asL1 embryos arrested at the four- and eight-cell stages in comparison with control, neg. siRNA and Neg. LNA embryos (71.61, 59.55 and 60.06 versus 41.78, 46.07 and 45.35%, respectively; *p* < 0.05; Table 2 and Supplementary Figure 3), and the rate of mimics group was significantly higher than that of inhibitors and LNA-asL1 groups (*p* < 0.05). These results show that whetherL1-siRNAs are overexpressed or knockdown leads to the failure of early embryonic development, suggesting L1-siRNAsplay regulatory roles in early embryonic development.

**Table 2:** Effect of L1-specific endo-siRNAs on *in vitro* development of porcine IVF embryos

Groups	Repeats	Embryos	Cleavage (%)	Blastocyst (%)	Embryos arrested at the four- and eight-cell stages (%)
**Con**.	4	360	238 (66.08 ± 8.67)	56 (16.57 ± 6.28)a	150 (41.78 ± 7.07)a
**mimics**	4	360	228 (63.28 ± 6.76)	16 (2.24 ± 2.24)bc	258 (71.61 ± 5.64)b
**Inhibitors**	4	360	222 (61.62 ± 8.58)	28 (8.51 ± 4.86)b	257 (59.55 ± 5.38)c
**Neg. siRNA**	4	360	254 (70.42 ± 9.46)	49 (14.27 ± 7.37)a	165 (46.07 ± 6.21)a
**LNA-asL1**	4	360	232 (64.32 ± 7.22)	19 (6.08 ± 3.73)b	217 (60.06 ± 3.36)c
**LNA-L1**	4	360	251 (69.57 ± 9.33)	10 (1.42 ± 1.42)c	275 (76.55 ± 7.97)b
**Neg. LNA**	4	360	242 (67.53 ± 8.42)	61 (17.35 ± 5.36)a	163 (45.35 ± 3.21)a

Note: values with different superscripts in the same group differ significantly (*p* < 0.05).

### L1-siRNAs control L1 retrotransposition during early embryonic development

**Figure 4:**
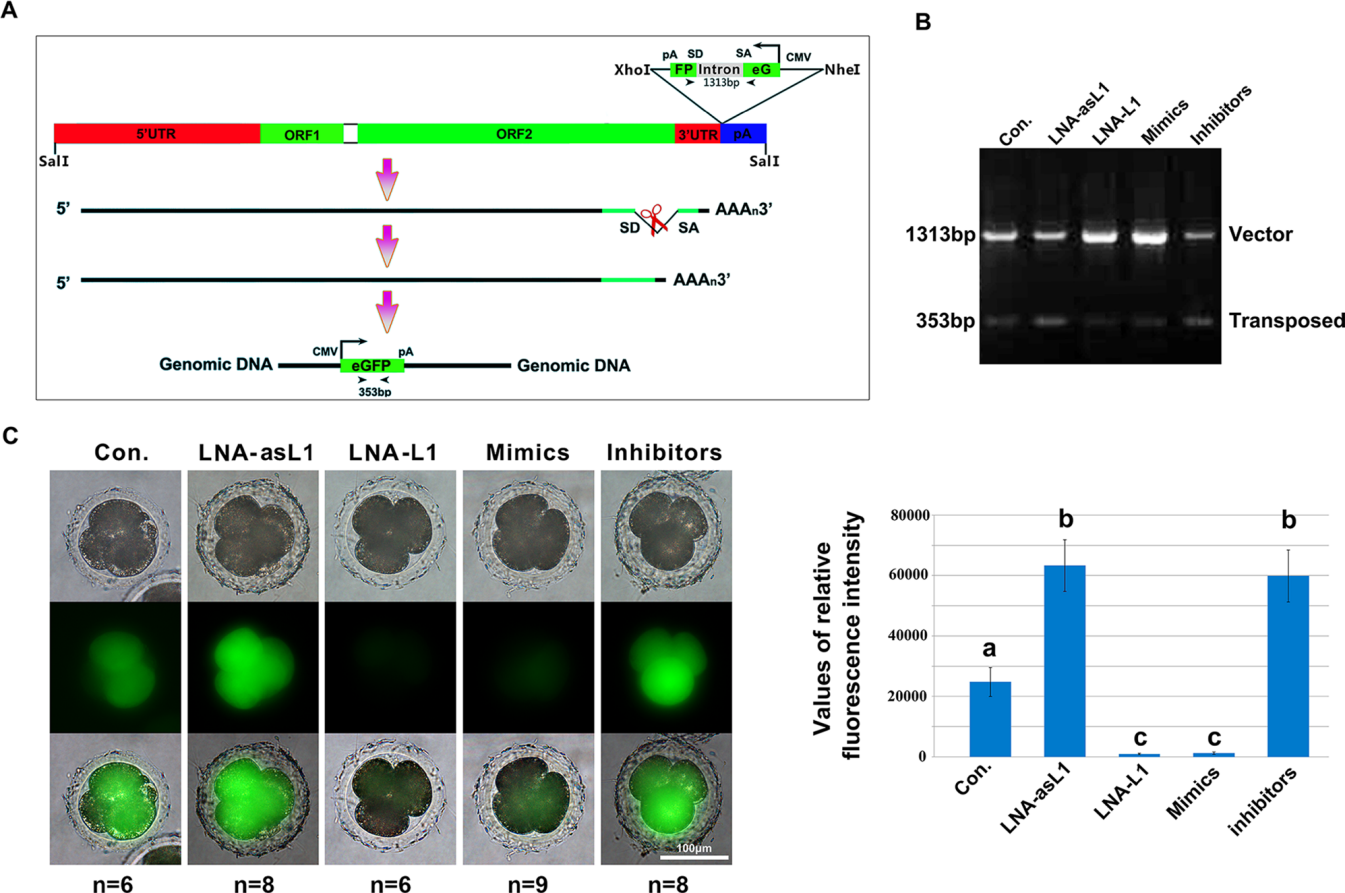
L1-siRNAs control L1 retrotransposition (**A**) Schematic diagrams of the pCEP4-pL1-eGFP expression cassettes used for L1 retrotransposition assays. This L1 retrotransposon contains an intron-interrupted eGFP reporter in the 3′ UTR region with its own CMV promoter and polyadenylation signal. The eGFP indicator cassette is in an antisense orientation relative to L1. Only when eGFP is transcribed from the L1 promoter, spliced, reverse transcribed and integrated into the genome does embryo become eGFP positive. Arrowheads depict the primers used in PCR based genomic DNA analysis. SD, splice donor; SA, splice acceptor; (**B**) PCR analysis of retrotransposition events in 4-cell stage embryos. The primers, flanking the intron in eGFP, were used for PCR amplification of genomic DNA, and the obtained PCR products of 1313 bp (corresponding to the intron-containing vector) and 353 bp (corresponding to the retrotransposed insertion that lacks the 960 bp intron) are shown; (**C**) Fluorescence detection of retrotransposition events in 4-cell stage embryos. Error bars represent s.d.. Values with different superscripts differ significantly (*p* < 0.001).

Previous reports have evidenced that L1 retrotransposition is strictly required in mouse early embryonic development [28, 29]. Definitely, we found L1 knockdown by LNA-L1 significantly decreased the rate of embryos developed to blastocysts and significantly increased the rate of embryos arrested at the four- and eight-cell stages compared to other groups (*p* < 0.05; Table 2). Moreover, endo-siRNAs can repress their targets by RNAi, so we hypothesize that L1-siRNAs may regulate early embryonic development by regulation of L1 retrotransposition. To clarify the mechanism, we firstly detected the expression of L1, asL1 and L1-siRNAsduring porcine early embryonic development. Q-PCR analysis showed the expressions of L1 ORF1 and ORF2 were enhanced from zygotes to the four- and eight-cell stages and were at the lowest levels at the blastocyst stage (*p* < 0.001; Figure 3A). And, the expression of asL1 and L1-siRNAs were enhanced to the highest levels at the four- and eight-cell stages (*p* < 0.001) and, the expressions, at the blastocyst stage, were also significant higher than that in zygote and two-cell stage (*p* < 0.001; Figure 3B and 3C). Further, LNA-L1, LNA-asL1, L1-siRNAs mimics and inhibitors were injected into MII oocytes to check the expression changes of L1 at the 4-cell stage. We observed the expressions of L1 ORF1 and ORF2 significantly decreased by LNA-L1 and L1-siRNAs mimics and increased by LNA-asL1 and L1-siRNAs inhibitors (*p*< 0.001; Figure 3D). Then, the engineered porcine L1 retrotransposition cassette was injected into MII oocytes to create a retrotransposition assay that accurately detects endogenous L1 retrotransposition events (Figure 4A).In the assay, LNA-asL1 and L1-siRNAs inhibitors induced high retrotransposition events in 4-cell embryos, and, in contrast, low retrotransposition activity was detected by LNA-L1 and L1-siRNAs inhibitors (Figure 4B and 4C). These results suggest that L1-siRNAs regulate early embryonic development by regulation of L1 retrotransposition.

## DISCUSSION

Endo-siRNAs are widely used as main endogenous regulators of gene expression [4, 5]. However, it is safe to say that we do not understand the specific biological functions of endo-siRNAs well. The function of endo-siRNA remains mostly unanswered in worms, and the discovery of abundant endo-siRNAs in flies and mouse makes the understanding more urgent. Some studies do show deregulation of retrotransposon transcripts, pseudogene-complementary transcripts and some cis-NAT pairs in Dicer and/or Ago mutants [9–12], but the function of specific endo-siRNAs has been seldom studied. To obtain a comprehensive picture of endo-siRNAs, in the study, a comparative characterization of porcine sperms, oocytes and zygotes, based on a large number of scnRNAs identified by means of deep sequencing and bioinformatics analysis, was carried out. The sncRNA data in pig were seldomly reported, and in the study, miRNAs and piRNAs were identified mainly by homologous alignment, so there are a number of unidentified scnRNAs in unknown group. Endo-siRNAs were characterized according to the precursor structure of dsRNA in detail. In addition to a large amount of TEs-siRNAs identified, we described 9 mRNAs with 16 IC-siRNAs and 21 LhpRNA loci with 182 Lhp-siRNAs in porcine sperms, oocytes and zygotes (Supplementary Table 3). Lots of endo-siRNAs were basically the same exact sequence, indicating DICER may recognize the specific sites to cut dsRNAs. Whatever, the data allow us to deepen our insight into the biogenesis of endo-siRNAs and to clarify their contributions to early embryonic development.

Many studies have identified different classes of small RNAs in sperm, however, endo-siRNA expression has been only described in female germ cells [6, 7, 30, 31]. In the study, we have characterized endo-siRNAs in porcine sperm, suggesting endo-siRNAs have potential roles in both spermatogenesis and oogenesis. Moreover, we observed some maternally and paternally inherited endo-siRNAs were eliminated in zygote while at the same time some specific endo-siRNAs were highly expressed after fertilization. Those high-expressed endo-siRNAs in zygote that replace to the parental endo-siRNAs might participate in early embryonic development. Though it has been evidenced that sperm-borne endo-siRNAs are essential for early embryonic development in Dicer KO mice [12], the function of specific endo-siRNAs has not been demonstrated. To reveal the role for specific endo-siRNAs during embryonic development, we are interested in a cluster of high-expressed L1-siRNAs mapped to the ORF2 regions of L1 retrotransposon in zygotes. Hundreds or thousands of TEs are inferred to generate endo-siRNAs, but the precise structure of the dsRNA substrates of endo-siRNAs processed from TEs is unknown. Endo-siRNAs derived from 5′ UTR region of L1 retrotransposon have been demonstrated in human cells but from L1 ORF2 have not been observed [27]. Here, we firstly found a homologous sequence of L1 ORF2 at L1 upstream sequence on chromosome 2. The observation of homologous sequences of L1 ORF2 is not surprising, given that the vast majority of L1 copies are variably truncated and/or rearranged at the 5′-end [29, 32]. Furthermore, we showed the homologous sequence triggered by L1 ASP could produce an antisense transcript, asL1, and a dsRNA could be formed betweenasL1and transcript of L1 ORF2, and converted into 9 L1-siRNAs. Abundant antisense transcripts with potential regulatory functions are encoded by eukaryotic genomes [3, 33], but the regulatory mechanism is poorly understood. Here, we characterize a previously unidentified asL1, which generates L1-siRNAs with L1 transcriptional output. Our data help understand the mechanism of antisense RNAs in the regulation of sense transcripts.

Until recently, the function of specific endo-siRNAs has not been determined during early embryonic development. In the study, we observed that regardless of whether L1-siRNAs were up regulated (mimics injection) or down regulated (inhibitors or LNA-asL1 injection), the development to blastocyst stage of embryos was failed, indicating L1-siRNAs are essential for early embryonic development. In human cells, it has been shown that L1-encoded endo-siRNAs trigger an RNAi effect on L1 [27, 34]. Our data are consistent with the previous knowledge that L1-siRNAs regulate L1 expression and retrotransposition in porcine early embryos. L1 retrotranspositions are high in undifferentiated or poorly differentiated cells including embryos, gametes and transformed cells; in contrast, terminally differentiated cells show only a basal level [35]. In porcine embryos, evidences presented here demonstrated L1 expression was activated to the highest level at the four- and eight-cell stages during zygotic genome activation and reduced to the lowest level at the blastocyst stage, suggesting L1 may play a causative role during early embryonic development.

Indeed, previous reports have found that L1 retrotransposon is required for early embryo preimplantation development in mouse by providing reverse transcriptase activity, which is required for proliferation of blastomeres in early cleavage-stage embryos [28, 29].A high proportion of genome is made of L1 elements that can be transcribed but most of them have been rendered inactive through mutations. The high sequence variability of L1 mRNAs in the cells makes it challenging to knockdown the expression of most of L1 copies. However, consistent with early reports [28, 29], we found LNA-siRNA targeted to 5′ end of ORF1 could efficiently knockdown L1 expression and retrotransposition. In the study, we also observed L1 knockdown in porcine embryos resulted in low activity of retrotransposition and early development failure, and the developmental failure led by overexpression of L1-siRNAs mimics in early embryos can be explained. We also observed the developmental failure of L1-overexpressed embryos by asL1 knockdown and overexpression of L1-siRNAs inhibitors. The explanation could be that high activity of L1 retrotransposition in L1-overexpressed embryos may increase the frequency of L1 mobilization, resulting in disrupting genes and altering splicing sites, and negatively affect the stability of the genome. The high and ongoing L1 retrotransposition can cause embryonic development failure. Thus, we conclude that L1-siRNAs regulate early embryonic development by controlling L1 retrotransposon.

In summary, endo-siRNAs present in porcine sperms, oocytes and zygotes were identified in detail. L1-siRNAs generated from a dsRNA formed between L1 transcript and a newly identified asL1 were characterized. We show that an RNAi effect induced by L1-siRNAs can control L1 retrotransposition in porcine early embryos. The effect could be highly important to regulate early embryonic development in mammals. Our work can contribute to understanding the role of endo-siRNAs in gene regulatory pathways.

## MATERIALS AND METHODS

### Porcine sperms, oocytes and zygotes collections

Sperms acquisition, oocytes *in vitro* maturation and *in vitro* fertilization were performed as described [36]. 5 × 10^9^ sperms, 7845 zona pellucida-free oocytes and 6800 zona pellucida-free zygotes were stored in TRIzol^®^ Reagent (Invitrogen) and frozen at −80°C until use.

### Purification and sequencing of small non-coding RNAs

Total RNAs from sperms, oocytes and zygotes were isolated using TRIzol^®^ Reagent (Invitrogen) according to the instructions provided by the manufacturer. RNA concentrations were quantified by measuring absorbance (A260/280ratio) on a NanoDrop Spectrophotometer ND-1000 (NanoDrop). RNA integrity number (RIN) was assessed using an Agilent 2100 Bioanalyzer (Agilent Technologies). Illumina protocols (available on the Illumina website at www.illumina.com/support) were followed to prepare the small RNA libraries from total RNA. Briefly, 3′ and 5′ adapters were ligated to small RNAs (< 200 nt) isolated from total RNAs from sperms, oocytes and zygotes. After adapter ligation, an RT reaction was performed to cDNA synthesis. In order to avoid any bias, the cDNA was PCR amplified by using common primers that were designed against adapter sequences. After cDNA amplification, RCR products were isolated by gel purification and then sequenced by high-throughput deep sequencing using an Illumina^®^ Hiseq2000 sequencer.

### Annotation of endo-siRNAs, miRNAs and piRNAs

Raw sequence data in fastq format were filtered to remove reads with unknown nucleotides. Adaptor sequences were removed from the 3′ end of reads. The trimmed reads with < 18 nucleotides or > 30 nucleotides were discarded. These reads were further filtered to remove reads with less than or equal to two counts. The filtered reads were aligned to the pig genome (susScr3, released in Aug. 2011) using bowtie (http://bowtie-bio.sourceforge.net/index.shtml) with a perfect match criterion (−k1 -n0). The reads that were not mapped to the genome were discarded. For miRNAs identification, we downloaded the sequences from the miRBase (http://www.mirbase.org/) and used bowtie to identify the miRNA sequences with the perfect match criterion. The piRNA population were annotated by searching homologous sequences to human and mouse piRNAs from piRNABank (http://pirnabank.ibab.ac.in/). The other known noncoding RNAs, including transfer RNA (tRNA), ribosomal RNA (rRNA), small nuclear RNA (snRNA), small nucleolar RNA (snoRNA),were identified by using the fRNAdb database (http://www.ncrna.org/frnadb/). The 18–24nt sncRNAs that were not annotated as known noncoding RNAs were considered as putative endo-siRNAs. We further identified endo-siRNAs associated with transposons. we downloaded the pig database of repeat sequence (http://genome.ucsc.edu/), and extracted sequences including “LTR”, ”SINE”, ”L1”, and ”ERV” from the database, and created a FASTA file. Then, we aligned the reads of sncRNAs to the FASTA file using bowtie with a perfect match criterion. The mapped reads are designated as TE-siRNAs. To identify long hairpin RNA-derived siRNAs, we searched the genome region compassing more than 15 unique sncRNA sequences within less than 10 kb of the genome. We extracted the regions of every clusters from the start to the end to predict RNA secondary structure using RNAfold (http://rna.tbi.univie.ac.at/), and we characterized the endo-siRNAs mapped in the predicted long hairpin RNA as Lhp-siRNAs. For the identification of endo-siRNAs derived from inverted complement mRNAs, we searched the complementary region between pig mRNA (downloaded from http://genome.ucsc.edu/) using the blastall, and the reads mapped to complement regions were annotated as IC-siRNAs.

### 5′ and 3′ RACE

Total RNA was extracted with the RNeasy Mini RNA kit (Qiagen) and the 5′ and 3′ RACE system (Roche) was used. The 5′ and 3′ RACE PCR products were inserted into the TA cloning system (Invitrogen) and sequenced. The primers used for 5′ RACE were: sp1ex (GTAAACTGGTGCAGCCACTATGG), sp2 (CACTCTTGGGCATCTATCCGGAC) and sp3 (CACATACACCCGCATGTTCATAGC). For 3′ RACE, the primers were sp4ex (CACCAGCAGTGCAGGAGGGTTC) and sp5 (GGAGGGTTCCCTTTTCTCCACAG).

### Embryo manipulations

To perform Dicer1, Drosha and Dgcr8 knockdown experiments, 10 pl of 10 μM LNA-Dicer1, LNA-Drosha or LNA-Dgcr8 (Exiqon) was injected into GV oocytes and the expressions were checked in MII oocytes or zygotes. 10 pl of 10 μM LNA-L1 and LNA-asL1 were injected into MII oocytes to check the knockdown efficiency and the *in vitro* developmental competency of IVF embryos. Endo-siRNA mimics used in the study are small, chemically modified double-stranded RNAs that mimic endo-siRNAs and enable siRNA functional analysis by up-regulation of siRNA activity. Endo-siRNA inhibitors are small, chemically modified single-stranded RNA molecules designed to specifically bind to and inhibit endo-siRNA molecules and enable siRNA functional analysis by down-regulation of endo-siRNA activity.9 L1-siRNAs mimics and inhibitors were designed and synthesized by Invitrogen and mixed by 1:1, respectively. 10 pl of 20 μM mimics mixture or inhibitors mixture was injected into MII oocytes to perform IVF and detect the embryonic development. The embryos were cultured in porcine zygote medium-3 at 39°C in 5% CO_2_ in air. The cleavage and blastocyst rates were assessed at 48 and 156 h after activation.

### Western blot

The procedure for Western blot has been described previously [22]. Antibodies against DICER (ab14601, Abcam) were used, and β-actin (A1978, Sigma) served as a loading control.

### Q-PCR analysis

Total RNA was extracted using the PureLink^TM^ RNA Mini Kit (Ambion) according to the manufacturer's instructions, and reverse transcription was used to generate cDNAs using the High Capacity cDNA Reverse Transcription Kit (Applied Biosystems). SncRNA was extracted using the mirVana^TM^ miRNA Isolation Kit (Ambion) according to the manufacturer's instructions, and detected using All-in-One^TM^ miRNA qRT-PCR Kit (Genecopoeia) according to the manufacturer's instructions. Real time PCR was performed using SYBRPremix Ex Taq^TM^ (TaKaRa) and the 7500 Real-Time PCR System (Applied Biosystems). The reaction parameters were 95°C for 30 s followed by 40 two-step cycles of 95°C for 5 s and 60°C for 34 s. All the primer pairs used to PCR amplification were shown in Supplementary Table 4. 18s rRNA and 5s rRNA were used as reference genes. Ct values were calculated using Sequence Detection System software (Applied Biosystems), and the amount of target sequence normalized to the reference sequence was calculated as 2^−ΔΔCt^.

### Retrotransposition assay

An EGFP retrotransposition cassette (pCEP4-pL1-eGFP) was injected into MII oocytes to perform IVF. The retrotransposition cassette contained a full-length porcine L1 retrotransposon tagged at its 3′ UTR with an antisense eGFP expression cassette. The eGFP gene was disrupted by a 960bp sequence of the γ-globin intron in the same orientation as the L1 transcript (Figure 4A). This arrangement ensures that functional eGFP expression occurs only after L1 retrotransposition event. That is, following L1 expression, γ-globin intron splicing, reverse transcription and insertion of a copy of L1 into the genomic DNA of the host cell. The L1 retrotransposition activity can be determined by a PCR based genomic DNA analysis and fluorescence microscopy of eGFP. Fluorescence microscopy was performed as described. The average fluorescence intensity of eGFP in each group of embryos was calculated by Image J. The primers used in PCR analysis were 5′CAGACAATCGGCTGCTCTGATGC3′ (forward) and 5′GCTCGCTCGATGCGATGTTTC3′ (reverse).

### Statistical analysis

Statistical analysis was performed using SPSS 13.0 for MicroSoft ™ Windows. Data are shown as the mean ± SD. One-way ANOVA was used to assess any differences between groups. The Duncan method was employed for pairwise comparisons, followed by a Bonferroni correction. *p* < 0.05 (two-tailed) was considered statistically significant.

## SUPPLEMENTARY MATERIALS FIGURES AND TABLES








