# Supplementary material for: Identification and characterization of L1-specific endo-siRNAs essential for early embryonic development in pig
Source: Oncotarget. 2017 Feb 19;8(14):23167–76. doi: 10.18632/oncotarget.15517 (PMC5410294; doi:10.18632/oncotarget.15517)
Supplement: Supplementary file 1 [file oncotarget-08-23167-s001.pdf]

Identification and characterization of L1-specific endo-siRNAs essential for early embryonic development in pig

Supplementary Materials

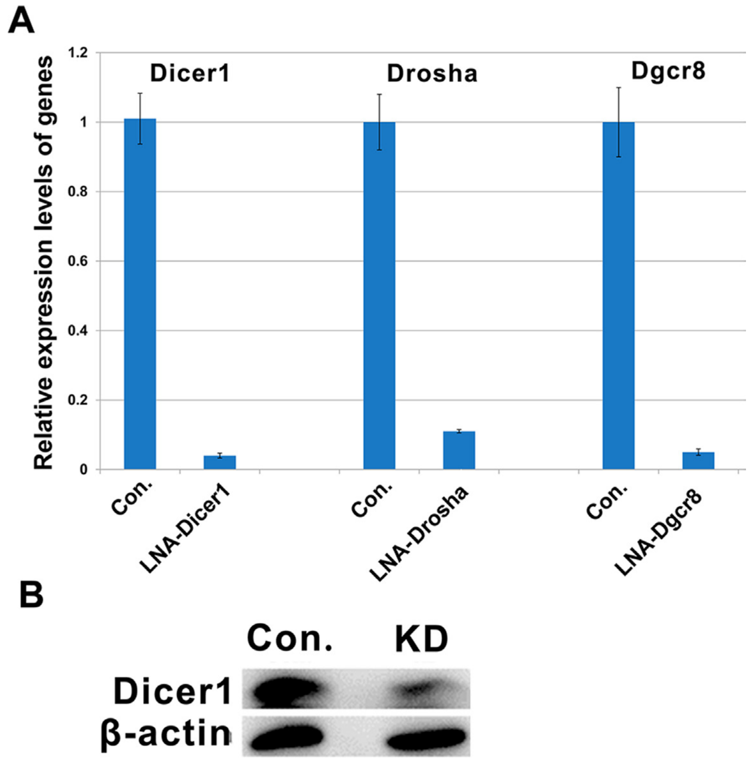

Supplementary Figure 1: Effective knockdown of Dicer1, Drosha, Dgcr8 by LNA-siRNAs. (**A**) Effective knockdown of Dicer1, Drosha, Dgcr8 mRNA checked by Q-PCR. Error bars represent s.d. (n = 3); (**B**) Effective knockdown of Dicer1 protein checked by Western blot.

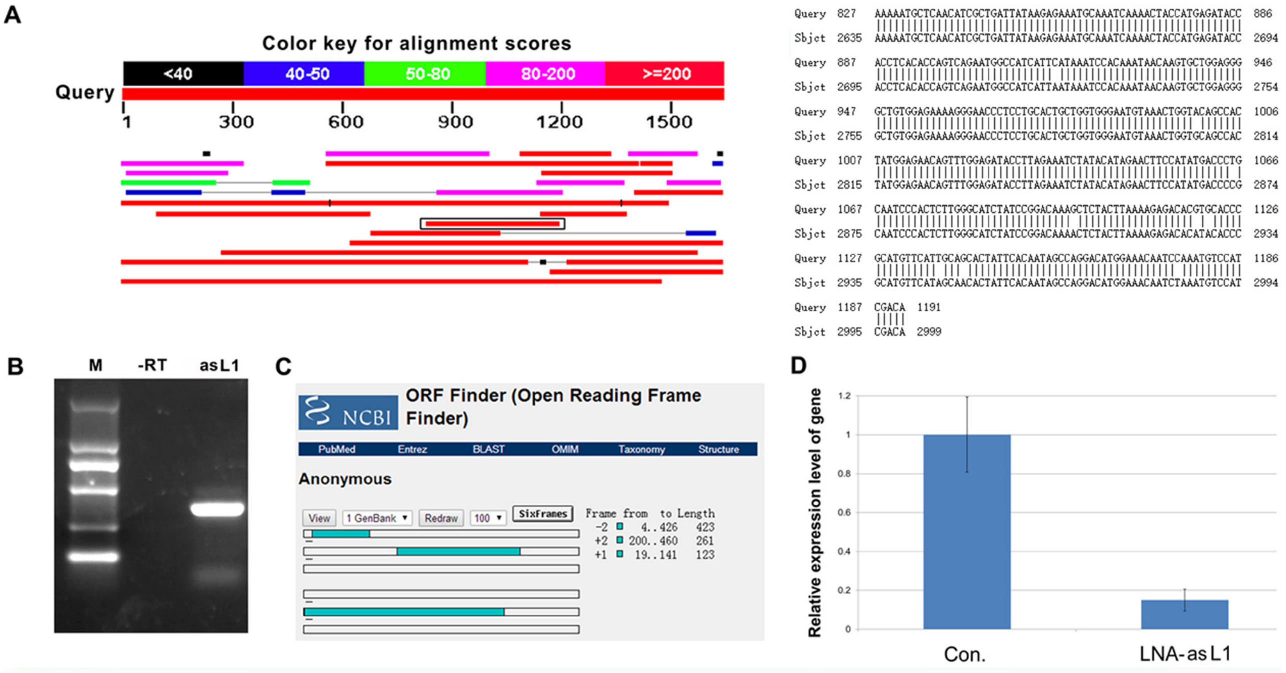

Supplementary Figure 2: Characterization of asL1. (**A**) Align ORF2 and 3' UTR sequences of L1 with upstream sequences of L1 using BLAST. The homologous sequence marked with black frame on chromosome 2 was further studied; (**B**) RT-PCR detects the expression of asL1. –RT, no reverse transcriptase; (**C**) Analysis of asL1 ORFs by ORF Finder; (**D**) Effective knockdown of asL1 by LNA-asL1. Error bars represent s.d. ($n = 3$).

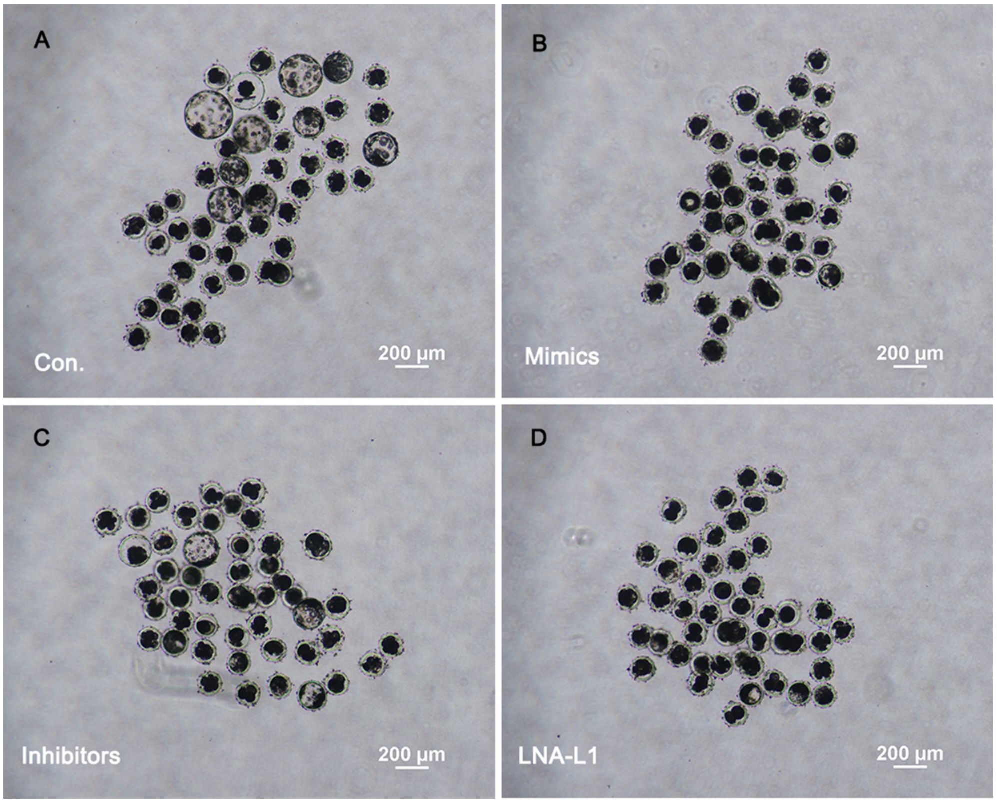

Supplementary Figure 3: Effect of L1-specific endo-siRNAs on *in vitro* development of porcine IVF embryos. (**A**) The embryos derived from uninjected oocytes (control); (**B**) The embryos derived from oocytes injected with L1-siRNAs mimics; (**C**) The embryos derived from oocytes injected with L1-siRNAs inhibitors; (**D**) The embryos derived from oocytes injected with LNA-L1.

Supplementary Table 1: Lists of piRNAs detected in porcine sperms, oocytes, and zygotes. See Supplementary_Table_1

Supplementary Table 2: Lists of miRNAs detected in porcine sperms, oocytes, and zygotes. See Supplementary_Table_2

Supplementary Table 3: Lists of endo-siRNAs detected in porcine sperms, oocytes, and zygotes. See Supplementary_Table_3

Supplementary Table 4: The primer list

Gene	Primer sequence (5′-3′)	Length (bp)	Accession number
18s rRNA	F: TCCAATGGATCCTCGCGGAA<br>R: GGCTACCACATCCAAGGAAG	149	NR002170
Dicer1	F: ACAAGATCCAGAGCTGGCTTA<br>R: CACAATGCTCGTGGCAATCA	172	NM_001197194.1
Drosha	F: GCCATCCCATGCTAGAACCT<br>R: CACTTCCGTGCCCTCCTTTAC	270	XM_013984710.1
Dgcr8	F: TCTATGAACTGACCAGCAAGGC<br>R: CCCAACTCGCTTATTCTTACACC	194	NM_001206919.1
L1 ORF1	F: GTGGGATTGCGGGGTCATATG<br>R: GTGCTGGAGGGGCTGTGGAG	140	EF599954.1
L1 ORF2	F: CAGCAAAGTGGCAGGATATAAGATTAAC<br>R:AATCCACAAATAACAAGTGCTGGAGG	106	EF599954.1
asL1	F: GGACATTTAGATTGTTTCCATGTCC<br>R: TGCTCAACATCGCTGATTATAAGAG	140	-
